# Population Dynamics and Life History Response to Precipitation Changes for a Desert Ephemeral Plant With Biseasonal Germination

**DOI:** 10.3389/fpls.2021.625475

**Published:** 2021-02-03

**Authors:** Xiao-Han Mu, Gang Huang, Yan Li, Xin-Jun Zheng, Gui-Qing Xu, Xue Wu, Yugang Wang, Yan Liu

**Affiliations:** ^1^State Key Laboratory of Desert and Oasis Ecology/Fukang Station of Desert Ecology, Xinjiang Institute of Ecology and Geography, Chinese Academy of Sciences, Urumqi, China; ^2^University of Chinese Academy of Sciences, Beijing, China

**Keywords:** ephemeral plant, precipitation change, biseasonal germination, population dynamics, life history

## Abstract

The changing availability of water resources and frequent extreme drought events in the context of global change will have a profound impact on desert vegetation, especially on herbaceous populations such as ephemerals. *Erodium oxyrrhynchum* is the dominant species in the Gurbantunggut Desert. It can germinate both in spring and autumn, which is important for herbaceous layer coverage and productivity. Therefore, we tracked and recorded the survival and reproduction of the *E. oxyrrhynchum* population under different precipitation treatments and established a population matrix model, monitored the allometry and leaf traits of the plants, and compared the performance of spring-germinating and autumn-germinating plants. Our results showed that: (1) The population dynamics were significantly affected by precipitation changes; (2) drought reduced the survival rate of the plants and accelerated the completion of their life history; (3) precipitation had a significant effect on seed production and growth rate, but not on plant height and allometry; (4) biomass, leaf area, specific leaf area, and 100-grain weight of *E. oxyrrhynchum* also responded to changes in precipitation; and (5) autumn-germinated plants had higher productivity, whereas spring-germinated plants exhibited higher reproductive efficiency, indicating that they had difference life history strategies. In conclusion, our results suggested that, although frequent or prolonged drought can significantly inhibit population growth, species with biseasonal germination are likely to be less affected.

## Introduction

Water availability plays an important role in the distribution and composition of plant communities (Schwinning and Ehleringer, 2001; Fay et al., 2003). Groundwater and precipitation are the main water sources available to plants in desert ecosystems, and the survival and distribution of shallow-root herbs are strongly dependent on precipitation. In the desert, the total precipitation is very low, and the precipitation pattern is variable and difficult to predict (Noy-Meir, 1973). Therefore, surface water availability and the survival of plants that depend on it are severely limited (Schlesinger et al., 1987; Wang et al., 2014; Zhu et al., 2014). Changes in precipitation caused by climate change will almost certainly affect the availability of water in deserts (Donovan and Ehleringer, 1994; Dai, 2013). The fluctuation of water content in shallow soil due to precipitation changes, coupled with the accelerated depletion of soil water caused by high temperature and evaporation, renders shallow-rooted desert plants vulnerable to water stress (Padilla and Pugnaire, 2007).

The Gurbantunggut Desert, located in Central Asia, it is an important habitat for ephemeral plants (Yang et al., 2019). Ephemeral plants are widely distributed and are the main contributors to plant biodiversity in this desert environment (Pan and Huang, 1995; Zeng et al., 2016). April to June is when the wind is strongest in the Gurbantunggut Desert (Wang et al., 2003). During this period, snowmelt and rainfall provide the necessary water for ephemeral plants to germinate and grow quickly. Consequently, the abundance of ephemeral plant cover is of great significance for stabilizing the sand surface (Wang et al., 2004; Li et al., 2009), and is thus vital for protecting neiboring agricultural land from sand invasion. However, ephemeral plants are highly dependent on, and sensitive to, spring precipitation (Wang et al., 2004, 2006; Yuan and Tang, 2010), and the uncertainty of precipitation in desert areas and global climate change both pose a challenge to ephemeral plant development.

In arid areas, water conditions are particularly important for the evolution of vegetation patterns (Zhao and Cheng, 2002; Jenerette et al., 2008). Given their importance for desert ecosystems, attention has increasingly been focused on ephemeral plants (Lu et al., 2011, 2014; Long et al., 2012). In the context of climate change, studies have been carried out on the effects of precipitation change and nitrogen deposition on the productivity of ephemeral plants (Fan et al., 2014; Huang et al., 2017a). However, population responses to climate change in this region have not received the deserved attention, especially those associated with ephemeral populations, that are likely to be among the most sensitive to climate change.

The herbaceous plant, *Erodium oxyrrhynchum*, is an important ephemeral plant in the desert community (Wang et al., 2003; Jia et al., 2018). The relative frequency of *E. oxyrrhynchum* in the Gurbantunggut Desert is >60%, while its importance value is 27, making it the dominant species in this desert (Zhang and Liu, 2012). As with other ephemeral plants in the desert, *E. oxyrrhynchum* can germinate again in the autumn at the end of the growing season if the rainfall is abundant (Chen et al., 2008). Seedlings that germinate in the fall will be covered by snow and will only be able to grow and reproduce in the spring (Zhang and Chen, 2002; Zeng et al., 2011). Having two germination seasons is a life history strategy that ephemeral plants have developed in response to the extremely variable desert climate (Evans et al., 2005; Simons and Johnston, 2006). Therefore, selecting *E. oxyrrhynchum* as the experimental species has broad significance for the whole local community.

Population dynamics can provide an overview of how ephemeral plants might respond to changes in climatic conditions. Matrix models are one of the most helpful tools in ecology, and are widely used in conservation biology, invasion biology, and population management research to assess population growth and viability of population structure (Leslie, 1945; Lefkovitch, 1965; Ellis et al., 2012). Hence, these models can be used to understand how population growth and life history respond to changes in climate, and what their life history strategies are (Crone et al., 2011). Here, we combined precipitation control, the monitoring of leaf traits, and plant life history with the population matrix model to understand (1) the influence of precipitation changes on individual and population growth of *E. oxyrrhynchum*; (2) the effects of precipitation changes on the life history of *E. oxyrrhynchum*; and (3) the differences in responses between spring- and autumn-germinated plants.

## Materials and Methods

### Study Site

The study site is located at the southern edge of the Gurbantunggut Desert in the Junggar Basin, Central Asia. The climate here is temperate continental, with an annual average temperature of 6.2°C, and annual precipitation of ~160 mm (Xu et al., 2007). Snow cover starts in December and lasts until March of the following year. The snowmelt in spring is an important water source for plant germination and growth, and is critical for maintaining plant species composition and diversity (Zhou et al., 2009). There were 40 species of ephemeral plants in the study area. Most of the ephemerals were members of the Brassicaceae and Asteraceae (Zhang and Liu, 2012), while the most common species were *E. oxyrrhynchum*, *Alyssum linifolium*, *Malcolmia scorpioides*, and *Hyalea pulchella* (Fan et al., 2014; Huang et al., 2015).

### Experimental Design

Between 2005 and 2016, the average precipitation during the growth period of ephemeral plants (April to June) was 61.7 mm, the maximum was 86.9 mm (in 2016), and the minimum was 22.9 mm (in 2012). Studies have shown that there are differences in vegetation coverage and seed yield between dry and wet years (Sun et al., 2009; Fan et al., 2014). Therefore, a controlled precipitation experiment was set up to simulate the conditions of drought years and wet years and observe the growth of *E. oxyrrhynchum* populations.

A total of three experimental treatments were established, namely, a drought treatment, simulating a drought year (precipitation reduced by half); the control treatment (natural precipitation); and a wet treatment, simulating a wet year (precipitation increased doubled). Each treatment had five replicates consisting of five adjacent 10 × 10 m^2^ quadrats. For topographic reasons, the adjacent quadrats are about 3–5 m apart, the edges of the quadrats acted as buffer zones. Highly transparent plastic sheets were cut into rectangles of 10 × 0.3 m, evenly spaced on a 10 × 10 m^2^ iron frame, to intercept half of the precipitation. Each rectangle was folded in half at a 120° angle, while the entire frame was lowered in front and raised in the back to facilitate the flow of rainwater. The intercepted rainwater was drained through PVC pipes to a collection tank. The collected rainwater was used to add the precipitation in the wet treatment whenever it rained.

The experimental site was located in an area of uniform plant density and growth. In October 2016, a large number of germinated seedlings were found in the study site, and were marked as autumn-germinated (AG) seedlings and individually numbered. After approximately 1 month, the vegetation was covered with snow. In early April 2017, the snow in the desert melted away. On April 13, 2017, 1 week after the germination of ephemeral annual plants, spring-germinated *E. oxyrrhynchum* plants were marked as spring-germinated (SG) seedlings and individually numbered. Between 400 and 600 individuals were labeled as SG and 150–300 as AG in each treatment. The edges of the quadrats acted as buffer zones and were set aside for destructive sampling, such as for the estimation of biomass.

### Plant Height, Growth Rate, and Seed Production

On April 20, 2017, the first height measurement was made for each marked plant using a ruler. When measuring plant height, the plants were marked with a number to facilitate the follow-up monitoring of individual plants. Subsequently, the height of each plant (both AG and SG) was measured once a week until the plants died. This method was used to obtain the height and growth trend of each plant. The difference between consecutive measurements was taken as the plant’s growth rate. The reproductive growth of *E. oxyrrhynchum* began in mid-May. The capsule of *E. oxyrrhynchum* splits when the seed matures, and the seeds fall off easily, making it difficult to record seed production once the seed has matured. To record seed production more accurately, and given that each capsule contains five seeds, the number of capsules in each marked plant was counted before seed ripening (in late May). Multiplying the number of capsules in each plant by 5 yielded seed production per plant.

### Plant Leaf Area, Specific Leaf Area, Biomass, and Hundred-Grain Weight

In May 2017, when the plants had reached their peak height, five whole healthy plants of average height in each of the quadrats were collected from the destructive sampling area and taken to the laboratory in a portable icebox. After removing the dust, the leaves were picked off. These leaves were first scanned (Epson Perfection 2400 Photo, Seiko Epson, Nagano, Japan) and the leaf area calculated (Computer Imaging Analysis Software, CID Co., Logan, UT, United States). After drying at 75°C for 48 h, each plant was weighted using an electronic balance (OHAUS, NJ, United States) to obtain the total aboveground biomass. After calculating the leaf area and separately weighing the leaves, the specific leaf area (SLA) was calculated as SLA = leaf area/leaf mass. Ten plants were selected from the destructive sampling area to collect all their seed, and to measure the 100-grain weight of each treatment.

### Demographic Measurements

The marked plants were observed once a week from April to June in 2017, which is the growing season for ephemeral plants. Plant survival was recorded during the observation period, and plant life history was divided into seedling, flowering, and reproducing states. In late May, before the seed ripened, the seed number in each marked plant was counted. When the seeds had ripened, the seeds were collected in destructive sampling area and allowed to dry naturally in the laboratory to test the germination rate. Then, the plants completed their life cycle and began to die. In autumn 2017, the autumn-germinating seedlings reappeared in the plots. The number of AG seedlings was counted in early October and individually marked. The survival of the AG seedlings was counted in April 2018.

The dried seeds were kept in a refrigerator at 4°C for 1 month as a cold stimulus for germination (Yin et al., 2009). The seeds were then placed in a petri dish with filter paper soaked in deionized water. The dishes were placed in an artificial climate chamber. The temperature of the chamber was set at 10 or 20°C and was switched every 12 h to simulate the desert temperature during seed germination. During this period, the germination of the seeds was recorded daily and the filter paper was kept moist. Seeds identified as having germinated were immediately removed and counted. The experiment was stopped when there was no germination for 1 week, at which point the germination rate of the seeds was calculated.

In March 2018, before snowmelt, the soil in the destructive sampling area was taken to the greenhouse. After sieving out the impurities and seeds, the soil was added to the bottom of flowerpots. Then, soil from the surface layer (5 cm) of the quadrats was evenly spread on the surface of the flowerpots. The flowerpots we use are 15 cm * 30 * 15 cm * 15 cm cuboids. The flowerpots were subsequently placed in the greenhouse to ensure that they received enough light and water. We use ventilation to ensure that the temperature in the greenhouse is consistent with wild. Germination was recorded every day, and the confirmed seedlings were immediately removed. The experiment was stopped when no new seedling had germinated for 1 week, after which the soil seedbank was counted.

### Data Processing and Analysis

**Figure 1:**
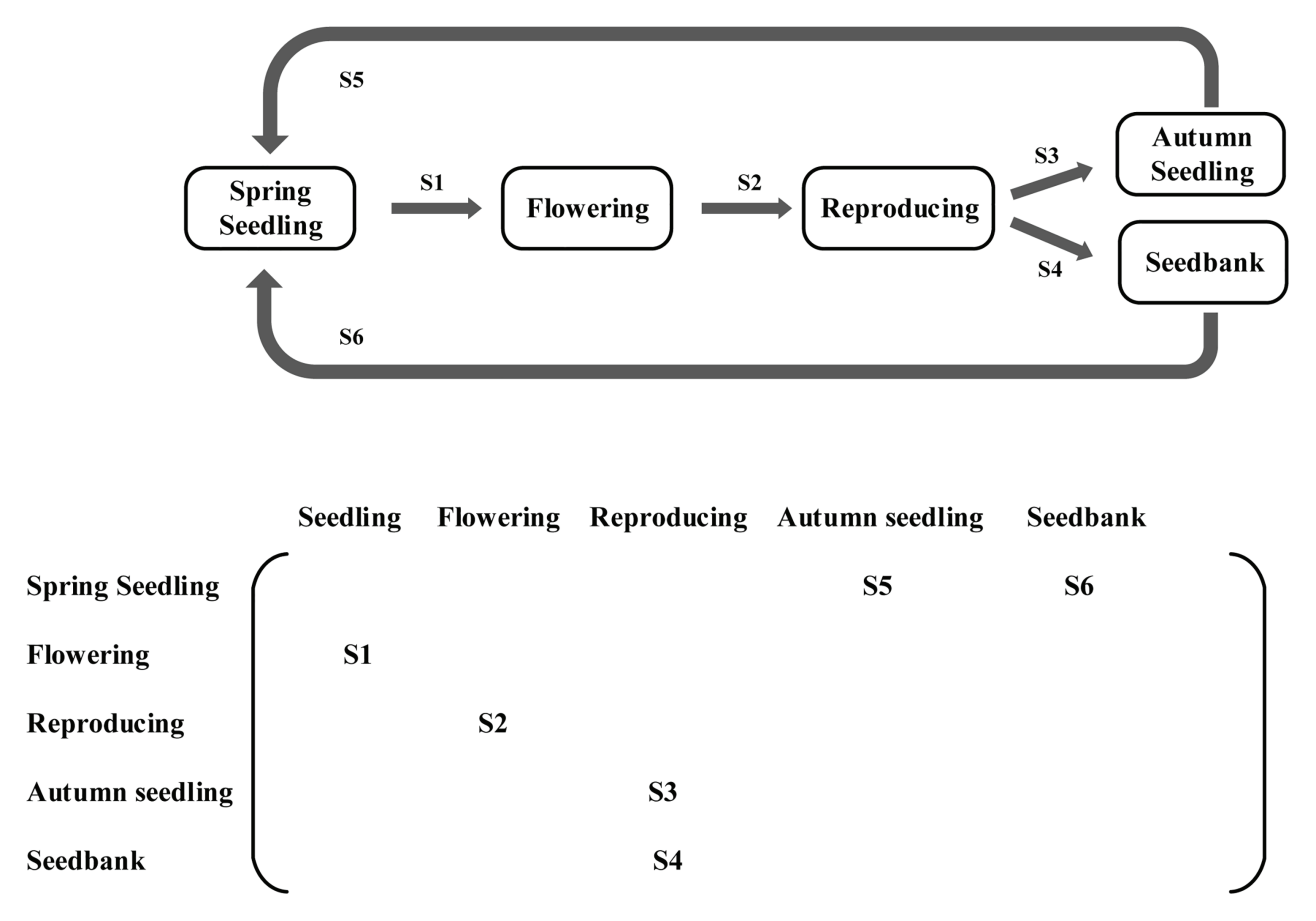
Life cycle and stage-classified matrix population model for *Erodium oxyrrhynchum* in the Gurbantunggut Desert from spring 2017 to spring 2018. The five stages in the model are spring seedling, flowering, reproduction, autumn seedling, and seed bank. The S1–S6 transitions are a probability of transition.

A stage-classified matrix model was used for five *E. oxyrrhynchum* life stages (Figure 1). The probabilities of S1, S3, and S5 transitioning were calculated using demographic censuses data from 2017 to 2018. The probabilities of S2 transitioning was calculated using the average seed production of *E. oxyrrhynchum* in each plot. The data from reproduction to seedbank (S4) were calculated from the soil seedbank experimental data obtained in March 2018. The germination rate was counted as the seedbank data for spring seedlings (S6) in each quadrat. The population growth rates (*λ*) were separately calculated in each plot as the dominant eigenvalues of the population matrix model (Caswell, 2001). To examine the importance of the different life stage transitions to population growth, elasticity analyses were also performed on average matrices from each treatment. The above analyses were conducted using the “popbio” package in R (Stubben and Milligan, 2007). Other data were analyzed by SPSS 21.0 (SPSS Inc., Chicago, IL, United States). We analyzed differences in plant height, growth rate, seed production, biomass, leaf area, specific leaf area, and 100-grain weight using a linear mixed model, in which germination time (SG or AG) and precipitation treatment (drought, control, or wet treatment) were set as fixed effects. The differences in plant height, growth rate, seed production, biomass, leaf area, specific leaf area, and 100-grain weight in each germination times among different precipitation treatments were analyzed using one-way ANOVA with Fisher’s tests. The differences in plant height, growth rate, seed production, biomass, leaf area, specific leaf area, and 100-grain weight between SG and AG at each precipitation treatments were processed using independent-sample *t*-tests. The differences in population growth rate among different precipitation treatments were analyzed using one-way ANOVA with Fisher’s tests. Survival analyses were carried out by the methods of Kaplan-Meier.We used standardized major axis (SMA) to analyze allometry. *p* < 0.05 was used to determine statistical significance. Figures were generated using Origin 8.5 software (OriginLab Corp., Northampton, MA, Unites States).

## Results

### Plant Height and Growth Rate

**Figure 2:**
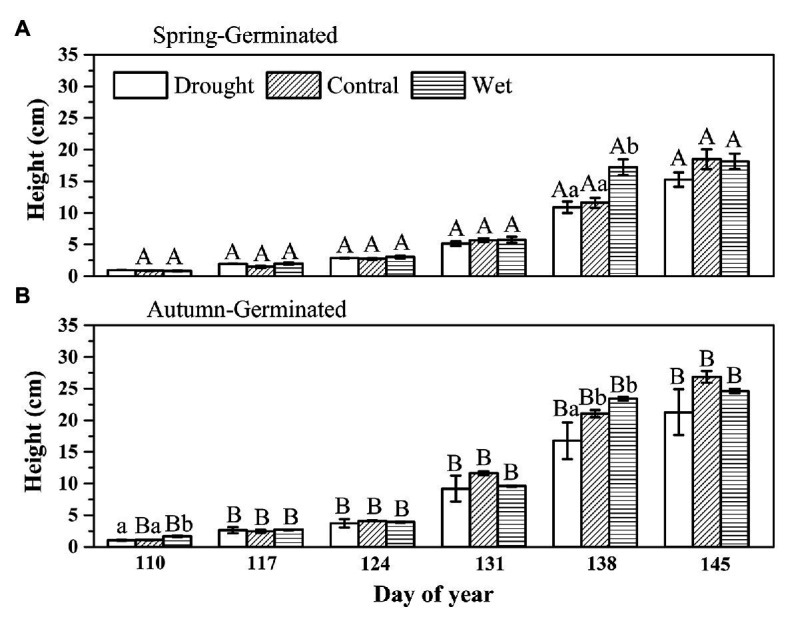
The height of spring-germinated **(A)** and autumn-germinated **(B)** *E. oxyrrhynchum* plants under drought, control, or wet treatment. For the drought treatment, the precipitation was reduced by 50%; for the wet treatment, the precipitation was increased by 100%. Different capital letters indicate significant differences between spring-germinated and autumn-germinated plants under the same treatments; different lowercase letters indicate significant differences between treatments (*p* < 0.05). The error bars represent the SEMs (*n* = 5).

The initial height of the plant is very small, and we used the initial plant height as the background value, because the precipitation treatments had not started at that time. The difference of initial AG height had no correlation with the final plant height and did not affect the final results. From day 110 to day 131, precipitation treatment had no significant effect on plant height for either SG or AG seedlings. On day 138, the height of SG seedlings under wet treatment was significantly greater than that under the other treatments (*p* < 0.05; Figure 2), while the height of AG seedlings under the drought treatment was significantly lower than that of other treatments. At day 145, precipitation treatment did not affect the height of either SG or AG seedlings (the final height of the plants). Nevertheless, the height of AG plants was always significantly higher than that of SG seedlings under all the treatments (*p* < 0.05; Figure 2).

**Figure 3:**
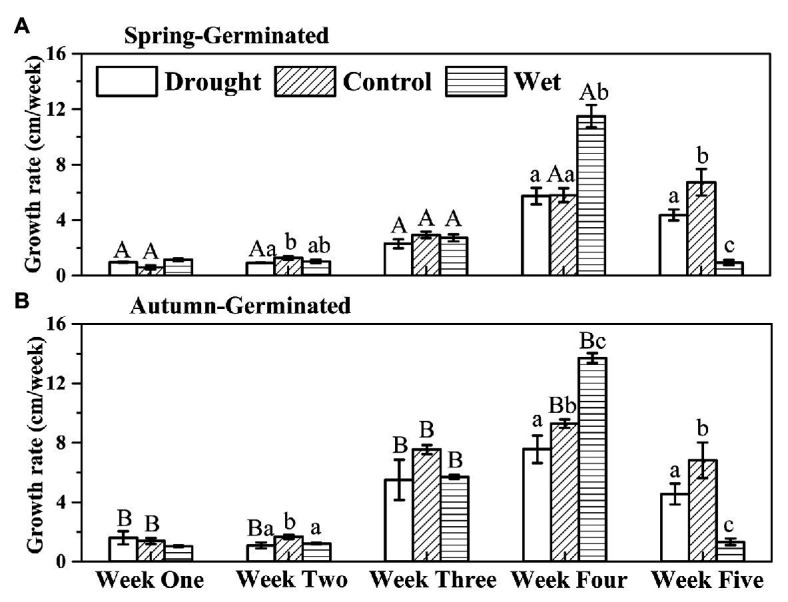
The growth rates of spring-germinated **(A)** and autumn-germinated **(B)** *E. oxyrrhynchum* plants under drought, control, or wet treatment. For the drought treatment, the precipitation was reduced by 50%; for the wet treatment, the precipitation was increased by 100%. Different capital letters indicate significant differences between spring-germinated and autumn-germinated plants under the same treatments; different lowercase letters indicate significant differences between treatments (*p* < 0.05). The error bars represent the SEMs (*n* = 5).

The height of SG plants increased slowly but steadily in the first 3 weeks; however, their growth rate began to accelerate from week 4, reaching a maximum (±SE) of 11.49±0.81 cm per week with the wet treatment (Figure 3). In week 5, the control and drought-treated SG plants exhibited sustained growth, whereas the growth of wet-treated plants was slow. For AG plants, the growth rate was slow in the first 2 weeks, but increased from the third week. In week 4, the growth rate (±SE) of the AG plants peaked at 13.70 ± 0.34 cm per week under wet treatment. In week 5, the growth rate decreased, but the plants continued to grow. The growth rate of plants undergoing wet treatment was significantly lower than that under other treatments. Nevertheless, the maximum growth rate of AG plants (13.70±0.34 cm per week) was greater than that of SG plants (11.49±0.81 cm per week); both maximum growth rates were recorded in week 4 with the wet treatment.

### Seed Production

**Figure 4:**
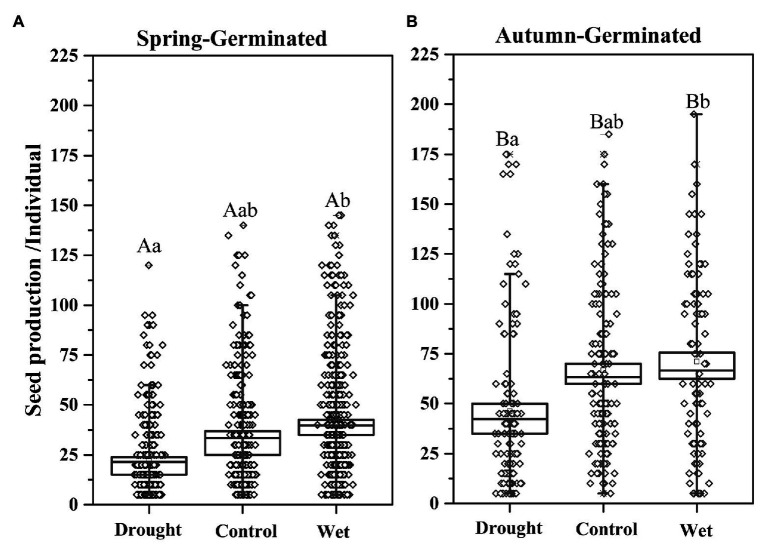
Seed production by spring-germinated **(A)** and autumn-germinated **(B)** *E. oxyrrhynchum* plants under drought, control, or wet treatment. For the drought treatment, the precipitation was reduced by 50%; for the wet treatment, the precipitation was increased by 100%. Different capital letters indicate significant differences between spring-germinated and autumn-germinated plants under the same treatments; different lowercase letters indicate significant differences between treatments (*p* < 0.05). The error bars represent the SEMs (*n* = 5).

Precipitation and the germination time had a significant effect on seed production (Figure 4). With the drought treatment, the average seed production (±SE) of SG plants was 22.00 ± 2.59; for the AG plants undergoing the same treatment, the average seed production was 50.20 ± 15.92, more than double that of the SG plants. Seed production (±SE) by both SG and AG plants was significantly higher with wet treatment (45.90 ± 3.86 and 71.83 ± 1.59 for SG and AG plants, respectively) than with the drought or control treatment. The average seed production by AG plants was significantly higher than that of SG plants (*p* < 0.05; Figure 4).

### Biomass, Leaf Area, Specific Leaf Area, and Hundred-Grain Weight

**Figure 5:**
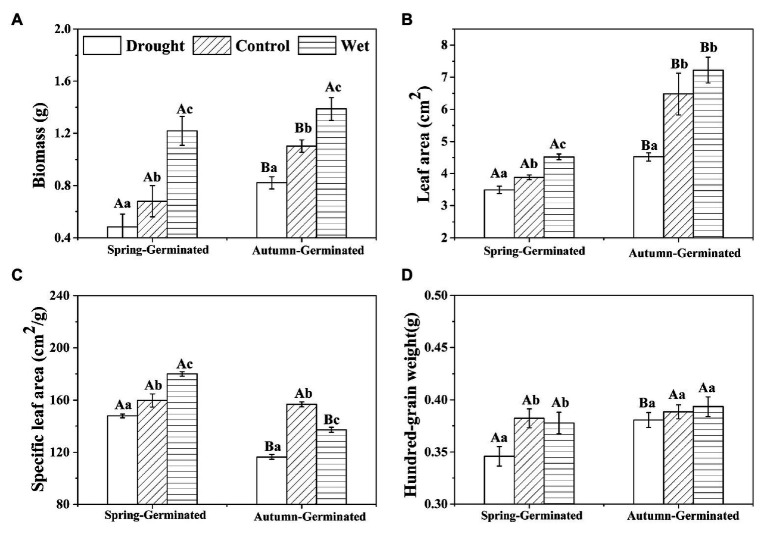
The biomass **(A)**, leaf area **(B)**, specific leaf area **(C)**, and hundred-grain weight **(D)** of spring-germinated and autumn-germinated *E. oxyrrhynchum* plants under drought, control, or wet treatment. For the drought treatment, the precipitation was reduced by 50%; for the wet treatment, the precipitation was increased by 100%. Different capital letters indicate significant differences between spring-germinated and autumn-germinated plants under the same treatments; different lowercase letters indicate significant differences between treatments (*p* < 0.05). The error bars represent the SEMs (*n* = 25).

The effect of precipitation on plant biomass was significant (*p* < 0.05; Figure 5). With increasing precipitation, the biomass (±SE) of SG plants gradually increased from 0.48 ± 0.03 g in the drought treatment group to 0.68 ± 0.04 g in the control group and 1.28 ± 0.09 g in the wet treatment group. Meanwhile, the biomass of AG plants increased from 0.82 ± 0.05 g in the drought treatment group to 1.10 ± 0.05 g in the control group and 1.39 ± 0.09 g in the wet treatment group. In addition, the biomass of AG plants was significantly greater than that of SG plants for all treatments (*p* < 0.05).

Precipitation had a significant effect on leaf area and specific leaf area of the plants. Wet treatment significantly increased the plant leaf area. The leaf area of AG plants was also significantly greater than that of SG plants in all treatment groups. With increasing precipitation, the specific leaf area of SG plants increased significantly (*p* < 0.05; Figure 5). However, for AG plants, the specific leaf area in the control group was significantly greater than that in the other treatment groups, and was smallest with drought treatment. Under drought treatment, 100-grain weight of SG was significantly decreased, and 100-grain weight of AG was significantly higher than SG.

### Kaplan-Meier Survival Analysis

**Figure 6:**
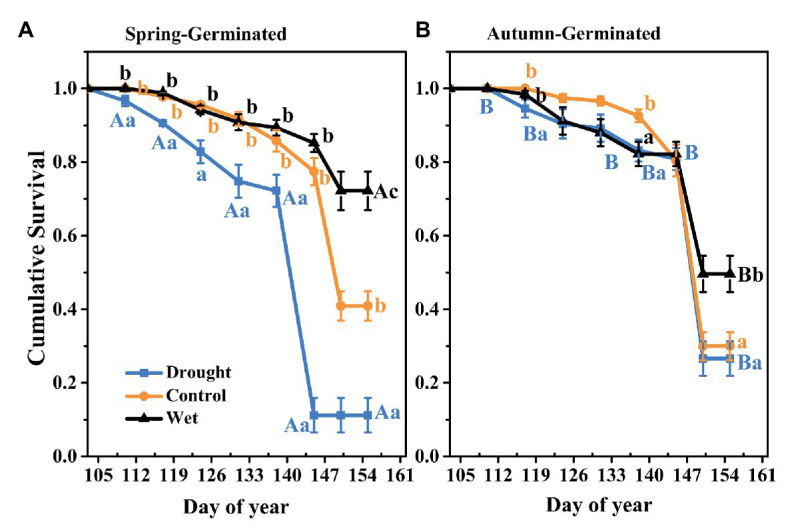
Kaplan-Meier survival analysis of spring-germinated **(A)** and autumn-germinated **(B)** *E. oxyrrhynchum* plants under drought, control, or wet treatment. For the drought treatment, the precipitation was reduced by 50%; for the wet treatment, the precipitation was increased by 100%. Different capital letters indicate significant differences between spring-germinated and autumn-germinated plants under the same treatments; different lowercase letters indicate significant differences between treatments (*p* < 0.05). The error bars represent the SEMs (*n* = 5).

For SG plants, Kaplan-Meier survival analysis showed that plant death was significantly greater with drought treatment than with the other treatments (*p* < 0.05; Figure 6) throughout the experiment. On day 145, there was a significant difference in plant survival rates among all the treatments. The survival of drought-treated plants was 11.2 ± 0.05%, whereas the rates with the control and wet treatments were 40.9 ± 0.04 and 72.2 ± 0.05%, respectively. For AG plants, the survival in the drought treatment group (26.6 ± 0.06%) and the control group (29.90 ± 0.04%) were significantly lower than that in the wet treatment group (49.6 ± 0.05%; *p* < 0.05; Figure 6). Meanwhile, with drought treatment, the survival rate of AG plants was greater than that of SG plants, whereas the opposite was observed with wet treatment.

### The Population Growth Rate

**Figure 7:**
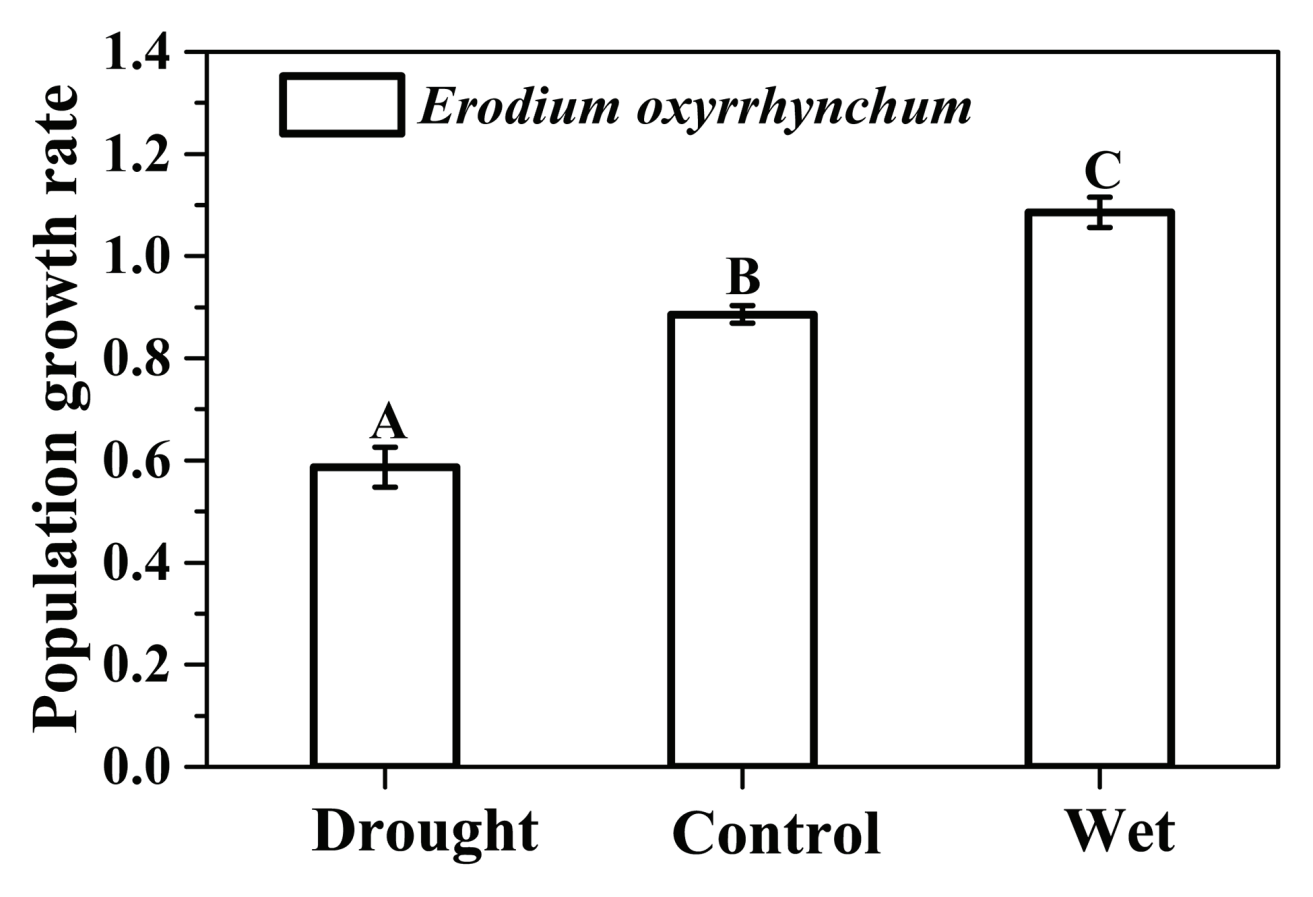
The population growth rate of *E. oxyrrhynchum* under drought, control, or wet treatment. For the drought treatment, the precipitation was reduced by 50%; for the wet treatment, the precipitation was increased by 100%. Different capital letters indicate significant differences between treatments (*p* < 0.05).

Linear-mixed model analysis revealed that precipitation significantly affected the population growth rates of *E. oxyrrhynchum* (Figure 7). With increasing precipitation, the population growth rate (±SE) increased from 0.58 ± 0.04 in the drought treatment group to 0.88 ± 0.02 in the control group and 1.08 ± 0.03 in the wet treatment group (*p* < 0.05; Figure 7).

### The Elasticities of Population Growth Rates

**Figure 8:**
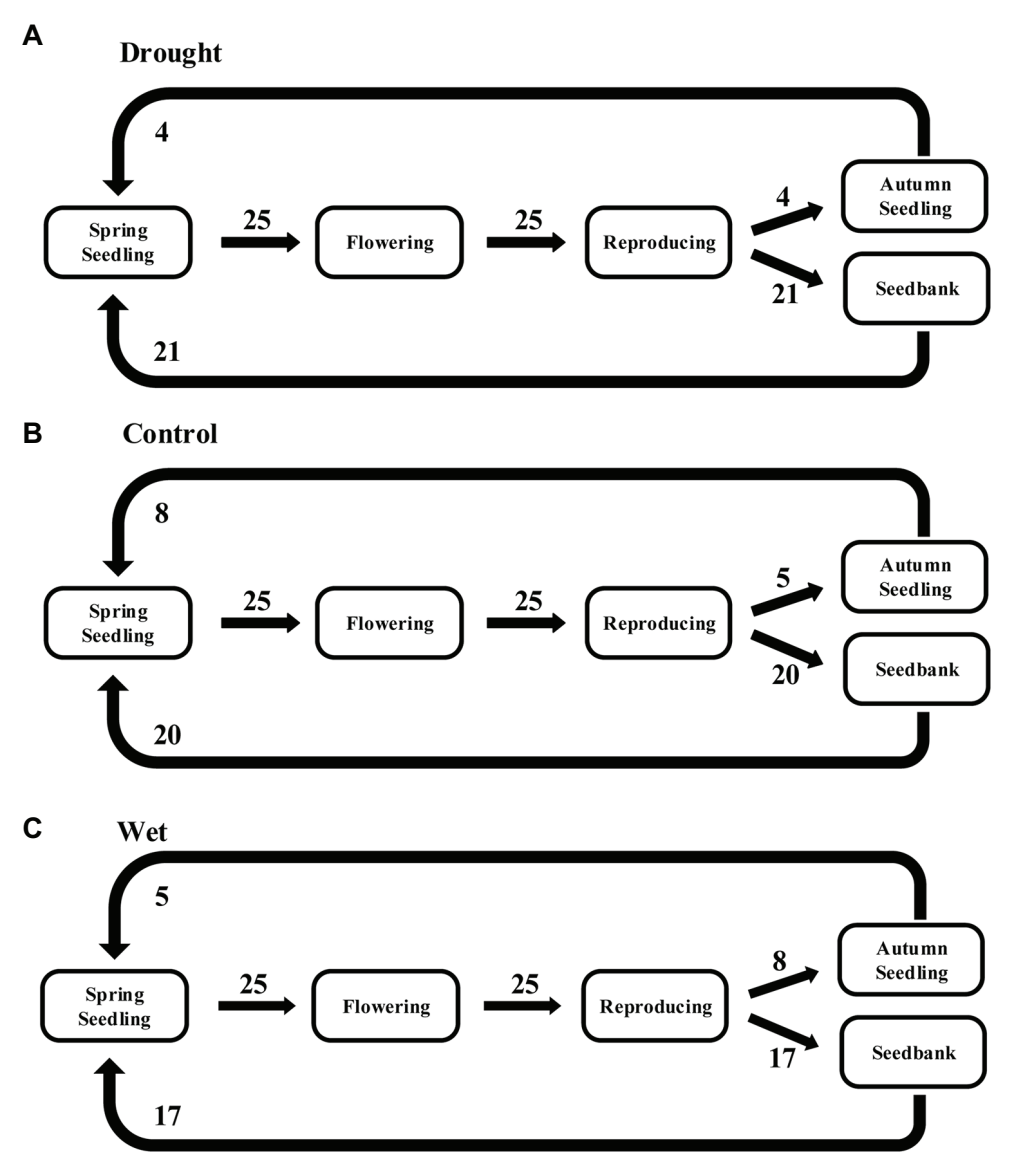
The elasticities of population growth rates of *E. oxyrrhynchum* under drought **(A)**, control **(B)**, or wet **(C)** treatment. For the drought treatment, the precipitation was reducedd by 50%; for the wet treatment, the precipitation was increased by 100%.

The results of the elasticity analyses revealed that the survival of spring-seedling to flowering and reproducing stages contributed more to population growth than the other transitions under all treatments, and was not affected by changes in precipitation. The germination and survival of the AG plants contributed the least to the population, and the transitions of AG plants that was affected most by precipitation. With increasing precipitation, the contribution of AG plants to population growth also increased (Figure 8).

### The Correlation Between Seed Production and Plant Height

**Figure 9:**
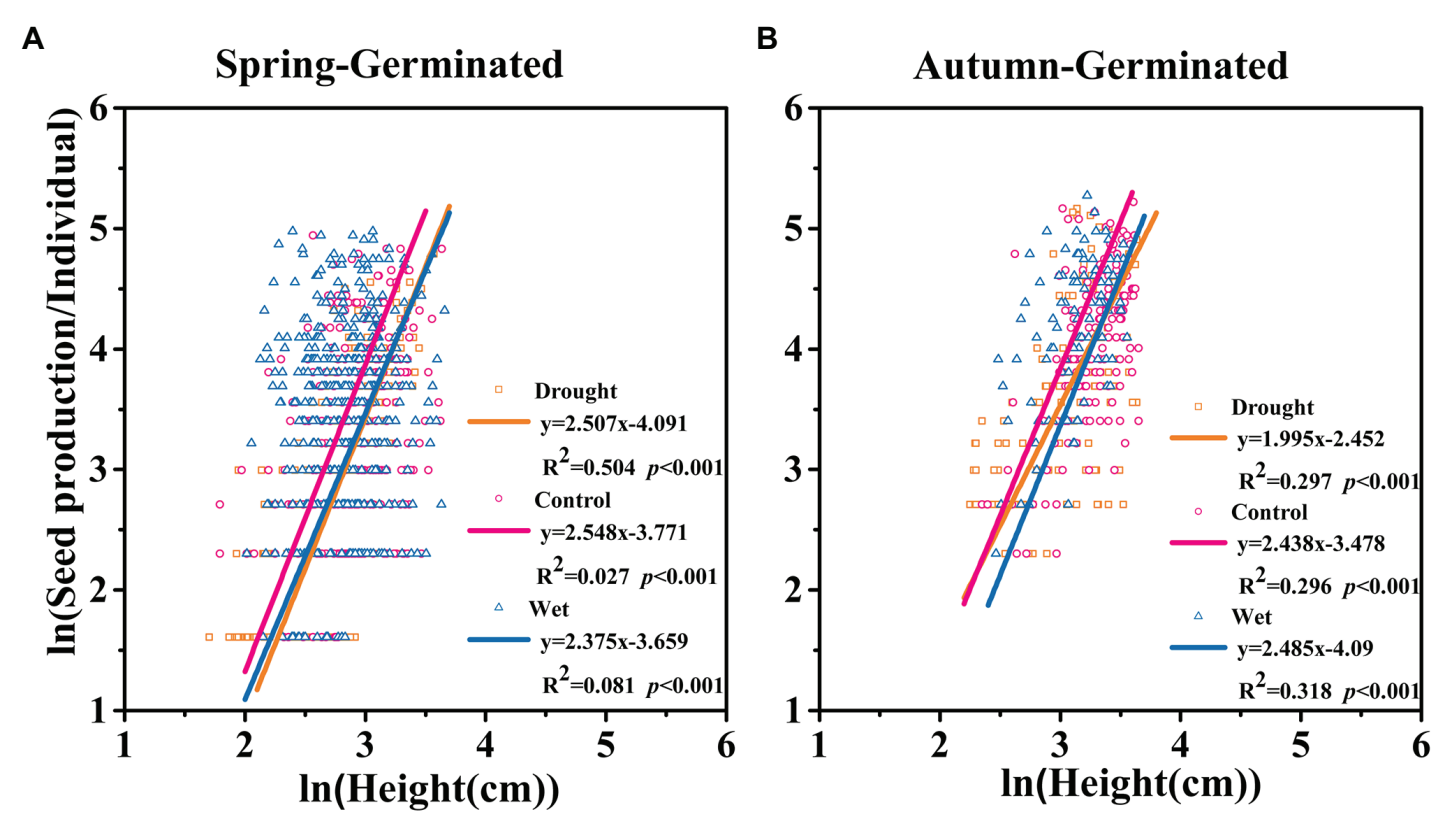
The correlation between seed production and height of spring-germinated **(A)** and autumn-germinated **(B)** *E. oxyrrhynchum* plants under drought, control, or wet treatment. For the drought treatment, the precipitation was reduced by 50%; for the wet treatment, the precipitation was increased by 100%.

Correlation analysis showed that the ln (seed production) of SG and AG plants was significantly and positively correlated with ln (height). However, precipitation did not significantly affect the slope of the curve, either for SG (*p* = 0.608, Slope_drought_ = 2.507; Slope_control_ = 2.375; Slope_wet_ = 2.548; Figure 9) or AG (*p* = 0.071, Slope_drought_ = 1.995; Slope_control_ = 2.485; Slope_wet_ = 2.438; Figure 9) plants. However, the slope was significantly different between SG and AG plants (*p* = 0.002; Figure 9). This indicated that AG and SG plants had different biomass distribution patterns. AG plants exhibited greater height and seed production, whereas SG plants had higher seed production at the same plant height.

## Discussion

Climate change will evoke changes in the life histories of herbaceous plants and their growth in arid areas (Prieto et al., 2008; Husen et al., 2017). In this study, we showed that precipitation had a significant effect on the population dynamics and population growth of *E. oxyrrhynchum*, and also affected its survival, life history, and leaf traits. SG and AG plants were both affected by precipitation, albeit to different degrees.

### Population Dynamics

Precipitation is essential for plant growth, relieving drought and promoting the establishment of plant seedlings (Donovan and Richards, 2000). In this study, precipitation changes had a significant effect on population growth (Figure 6), and a similar conclusion was reached in a previous study on the population dynamics of *Bromus tectorum*, namely, that increased precipitation in autumn and winter will render the species increasingly invasive (Prevey and Seastedt, 2015). The survival of seedlings in spring and their transitions to flowering and reproduction were the two most important transitions contributing to the population growth under all treatments (Figure 7). Seed production is the most important life history stage for herbs, and is essential for ensuring the reappearance of the population in the following year (Watkinson et al., 1989; Prevey and Seastedt, 2015).

Precipitation correlated well with the number of *E. oxyrrhynchum* AG plants, although it played only a small role in population dynamics (Figure 7). Previous studies have also shown that the occurrence of autumn germination and the density of AG plants were directly related to precipitation levels (Zeng et al., 2011). Our elasticity analyses indicated that the contribution of AG plants to population growth showed a gradual increase with increasing precipitation. These results further indicated that *E. oxyrrhynchum* adjusted its growth strategy through autumn germination to adapt to the climatic conditions.

The survival of herb plants is highly dependent on precipitation (Narita, 1998; Li et al., 2014). In the present study, increased precipitation significantly improved the survival rates of both SG and AG plants; however, AG plants were more tolerant to the drought treatment than SG plants (Figure 5). Previous studies had suggested that increasing precipitation levels could improve the survival of *E. oxyrrhynchum*, but significantly more so for SG plants than for AG plants (Chen et al., 2018). This was mainly due to the damage suffered by AG plants under the low temperature and arid environment in winter. We further confirmed this in a population dynamics observation in 2018. However, the AG seedlings that survived until spring had a longer vegetative growth period, which allowed for higher growth and survival rates compared with SG seedlings.

### Life History

Desert plants tend to show high plasticity in life history traits to cope with the unpredictability of their environment (Günster, 1994). Extensive studies have shown that plant phenology is affected by climatic changes such as increases in temperature, changes in precipitation, and levels of nitrogen deposition (Cleland et al., 2006; Xia and Wan, 2012; Gusewell et al., 2017). In desert ecosystems, the survival strategy of ephemeral plants involves rapid growth and life cycle completion when the climate is favorable, followed by rapid withering and survival as seeds under harsh conditions (Wang, 1993; Zhang and Chen, 2002). In our study, drought accelerated the completion of the life history of SG plants and changed plant phenology, which indicated that the plants had made adaptive changes to the changing environmental conditions (Parmesan and Yohe, 2003; Meineri et al., 2014). This illustrated that, although current global climate models predict that global change will increase the variability in precipitation (Fischer et al., 2013; Boeck et al., 2017; Huang et al., 2017b), ephemeral plants can elude unfavorable conditions by accelerating their growth and adjusting their life history.

The growth rates of the plants differed according to the growth stage, and the changes in precipitation affected their growth rates (Figure 2). This was likely due to the regulation of biomass allocation by the plants at different growth stages, which is an important environmental-adaptive strategy (Casper et al., 1998; Elsa et al., 2016). In the early stages of plant growth, more resources are allocated to root growth to help plants obtain more water and nutrients from underground resources, thereby helping them to cope with subsequent survival pressures and environmental changes and improve plant competitiveness (Yuan and Tang, 2010). Subsequently, in the middle stages of plant growth, more biomass is allocated to the aboveground portion of the plant for the growth of stems, leaves, and reproductive organs to achieve high fertility (Yao and Tan, 2005; McCarthy et al., 2007). Xiao et al. (2014) showed that the root to shoot ratio exhibited a decreasing trend during plant growth, which also demonstrated that desert herbs accumulate more biomass aboveground at the end of their growth to obtain enough photosynthetic products to complete sexual reproduction (Xiao et al., 2014). In our study, the wet treatment provided enough precipitation to greatly increase the growth rate of the plants, enabling their vegetative growth to be completed earlier than that observed with the other treatments (Figure 2). This is consistent with the results of a previous study on the phenology of desert plants, in which water addition enhanced the relative growth rate of the plants and altered their phenology (Huang et al., 2018). This indicates that the growth pattern of plants is an innate survival strategy, that adapt to the local environment, whereas a change in its growth rate represents an adjustment to climatic change.

Plant traits represent adaptations to the environment and trade-offs in a variety of functions (Yao et al., 2010). In our study, precipitation changes significantly affected the aboveground biomass, leaf area, and specific leaf area of the plants (Figure 4). In arid environments, plants usually display reduced biomass, leaf area, and specific leaf area, which helps them reduce their carbon consumption for building and maintaining stems and their transpiration, as well as improve their water-holding capacity (Falster and Westoby, 2003; Ma et al., 2006). Sufficiently wet conditions are conducive to the absorption of nutrients, plant growth, and increases in biomass accumulation (Shem-Tov and Gutterman, 2003). Larger leaf area can also increase the net photosynthesis of plants. These morphological adjustments are conducive to plant adaptation to the desert environment of drought, high temperatures, and intense radiation (Cunningham et al., 1999; Zhang and Luo, 2004). For AG plants, the specific leaf area decreased under wet treatment, which may be caused by phenological difference. Precipitation changes the life cycle of AG, leading to different resource allocation than other treatments.

In addition to water constraints, the effects of nutrient and density constraints on population growth also need to be considered, even in dry regions. Our results showed that precipitation exerted a significant influence on plant survival and seed production (Figure 4), but did not affect plant height (Figure 2) or resource allocation between vegetative growth and seed production (Figure 9). Similar results were reported for studies investigating the influence of water on the desert herb layer. Precipitation was shown to be positively correlated with plant density and vegetation coverage but negatively correlated with vegetation height; however, the species diversity of the community was not affected (Fan et al., 2014; Zhang et al., 2018). A different study showed that improved water availability could increase seed production by *Stipa krylovii* in Mongolia, but the seed viability was negatively correlated with precipitation (Ronnenberg et al., 2011). Adequate water not only benefits individual plant growth but also increases the density of the population. The productivity and available resources of ecosystems are limited, and a high population density intensifies competition for water, nutrients, and space among plants in a community (Enquist et al., 1998; Xiao et al., 2006). In summary, transient fluctuations in precipitation levels can affect plant survival, thereby determining the density of plants and population expansion without affecting population structure. Through their growth process, the population achieves relative stability *via* its own regulation.

### Comparison of Spring and Autumn Germinated Plants

Biseasonal patterns of germination can be regarded as a survival strategy that is influenced by the environment, and this strategy is important for seedling survival and plant fitness (Masuda and Washitani, 1992; Yang et al., 2019). In our study, AG plants had greater height (Figure 2), seed production (Figure 4), and aboveground biomass (Figure 5), as well as stronger resistance to spring drought, than SG plants (Figure 6); however, they were at higher risk of death and injury in the winter. SG plants had greater reproductive efficiency (Figure 8) and a higher survival rate, which explained the greater abundance of SG plants in the population. AG and SG plants each had their own advantages and shared the risks associated with environmental change, indicating that this strategy is conducive to the continuation and expansion of the population (Yao and Tan, 2005; Zhang et al., 2007). In addition, the biseasonal germination strategy dispersed the germination time, increased species fitness, and allowed the full use of the limited water resources in the desert (Li and Wang, 2003; Zeng et al., 2011). At the same time, the increase in precipitation event in autumn and winter (Piao et al., 2010) not only enhances the capacity of populations to withstand a changeable environment but also allows species with biseasonal germination strategies to occupy a dominant position in the community.

### Conclusion

The present findings contribute to the understanding of how changes in precipitation influence desert ephemeral plant populations and the adaptation strategies employed by these herbaceous plants under the background of global climate change. Under extreme drought, the survival and population growth rates were significantly inhibited. However, these ephemeral plants responded to the adverse conditions by speeding up their life cycle and reducing aboveground biomass, leaf area, and specific leaf area. When precipitation was abundant, their growth rate increased significantly, while their survival rate, population growth rate, and average seed production also increased. The contribution of AG plants to the population was also greater. In addition, irrespective of whether the environment was dry or wet, the population structure and the importance of seedling transitions to flowering plants for the population were not affected. SG and AG seedlings each had their advantages with respect to the population. These results suggested that the development of ephemeral plant populations is strongly dependent on precipitation; nonetheless, they exhibit high plasticity and adaptability to cope with the changeable environment. Moreover, biseasonal germination patterns provide additional advantages for ephemeral plants to adapt to a desert environment.

## Data Availability Statement

The original contributions presented in the study are included in the article/supplementary material, and further inquiries can be directed to the corresponding authors.

## Author Contributions

All the authors contributed to study conceptualization. GH conceived the study. X-HM conducted the experiments, analyzed the data, and drafted the manuscript. X-JZ and YLi critically reviewed and edited the manuscript. XW, YW, G-QX, and YLiu performed the experiments. All authors have read and approved the final version of this manuscript.

### Conflict of Interest

The authors declare that the research was conducted in the absence of any commercial or financial relationships that could be construed as a potential conflict of interest.
